# MONETA: A Processing-In-Memory-Based Hardware Platform for the Hybrid Convolutional Spiking Neural Network With Online Learning

**DOI:** 10.3389/fnins.2022.775457

**Published:** 2022-04-11

**Authors:** Daehyun Kim, Biswadeep Chakraborty, Xueyuan She, Edward Lee, Beomseok Kang, Saibal Mukhopadhyay

**Affiliations:** Department of Electrical and Computer Engineering, Georgia Institute of Technology, Atlanta, GA, United States

**Keywords:** spiking neural network (SNN), processing-in-memory (PIM), convolutional spiking neural network, on-line learning, on-chip learning, spike-time-dependent plasticity (STDP), AI accelerator, hybrid network

## Abstract

We present a processing-in-memory (PIM)-based hardware platform, referred to as MONETA, for on-chip acceleration of inference and learning in hybrid convolutional spiking neural network. MONETAuses 8T static random-access memory (SRAM)-based PIM cores for vector matrix multiplication (VMM) augmented with spike-time-dependent-plasticity (STDP) based weight update. The spiking neural network (SNN)-focused data flow is presented to minimize data movement in MONETAwhile ensuring learning accuracy. MONETAsupports on-line and on-chip training on PIM architecture. The STDP-trained convolutional neural network within SNN (ConvSNN) with the proposed data flow, 4-bit input precision, and 8-bit weight precision shows only 1.63% lower accuracy in CIFAR-10 compared to the STDP accuracy implemented by the software. Further, the proposed architecture is used to accelerate a hybrid SNN architecture that couples off-chip supervised (back propagation through time) and on-chip unsupervised (STDP) training. We also evaluate the hybrid network architecture with the proposed data flow. The accuracy of this hybrid network is 10.84% higher than STDP trained accuracy result and 1.4% higher compared to the backpropagated training-based ConvSNN result with the CIFAR-10 dataset. Physical design of MONETAin 65 nm complementary metal-oxide-semiconductor (CMOS) shows 18.69 tera operation per second (TOPS)/W, 7.25 TOPS/W and 10.41 TOPS/W power efficiencies for the inference mode, learning mode, and hybrid learning mode, respectively.

## 1. Introduction

Spiking neural network (SNN) (Maass, 1997; Gerstner and Kistler, 2002) with spike-time-dependent-plasticity (STDP) based unsupervised learning provides a bio-inspired and energy-efficient alternative to deep learning (Kim et al., 2020; Panda et al., 2020). There is a growing interest in developing specialized hardware accelerators for SNN (Akopyan et al., 2015; Buhler et al., 2017; Davies et al., 2018; Chen et al., 2019; Park et al., 2019; Chuang et al., 2020). However, majority of the prior accelerators focused on fully connected SNN and shallow networks. Deep Convolutional Neural Network (CNN) architectures incorporated within SNN, hereafter referred to as ConvSNN, can improve the accuracy of SNNs for complex problems (Cao et al., 2015; Tavanaei et al., 2016; Kheradpisheh et al., 2018; Lee et al., 2019). As the complexity of ConvSNN increases, deep ConvSNN requires more synaptic weights and generates larger input/output feature maps, all of which can increase data movement. Processing-in-memory (PIM) has emerged as a key approach to reduce data movement and enhance the energy efficiency of CNNs (Chi et al., 2016; Shafiee et al., 2016; Imani et al., 2019; Long et al., 2020; Sze et al., 2020). However, to the best of our knowledge, there has been no prior work on PIM based accelerator for ConvSNN with on-chip learning.

This article for the first time presents a PIM, hereafter referred to as MONETA, to accelerate ConvSNN with on-chip STDP learning. The overall architecture of MONETAincludes SRAM-based PIM cores for computing synapse responses, all-digital modules for computing membrane potentials of neurons, and centrally manage but locally apply STDP-based weight update. The SRAM-based PIM cores augment the sequential access PIM used in DNN acceleration, such as the ones presented by Long et al. (2020), with STDP-based weight update modules for parallel updates of synaptic weights (Kim et al., 2020). The novelty of MONETAlies in the optimized data flow for improving resource efficiency while implementing inference and learning in PIM-based ConvSNN.

**Figure 1:**
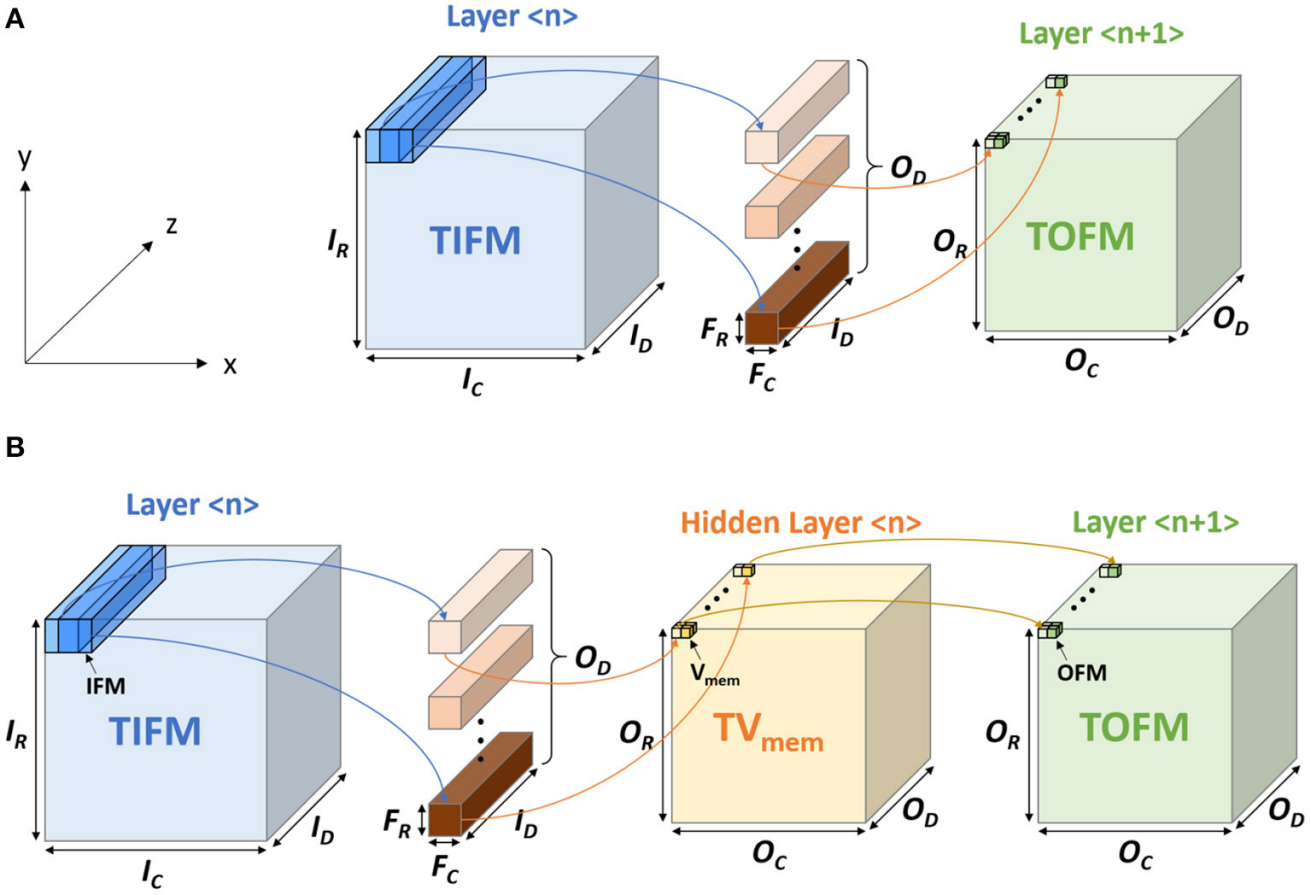
The computational model in **(A)** convolutional neural network (CNN) and the **(B)** convolutional neural network within spiking neural network (ConvSNN).

In traditional CNN, the output feature map (OFM) tensor of a layer is obtained from the total input feature map (TIFM) tensor and filter weights (Figure 1A). In ConvSNN, we first generate a tensor for the membrane potential of all neurons (*TV*_mem_), followed by output spikes (OFMs) (Figure 1B). However, as input pixels are encoded as spike trains, multiple time steps (spike cycles) are necessary to process one image using ConvSNN. Hence, the TIFM for each layer must be processed multiple times to generate the *TV*_mem_ in each spike cycle, leading to a large on-chip buffer for *TV*_mem_ tensor, and significant off-chip (from DRAM) and on-chip (from *TV*_mem_ buffer) data movement. Although, Narayanan et al. have analyzed the temporal aspects of SNN for logic-based engines (Narayanan et al., 2020), they did not optimize data flow simultaneously considering data movement and learning accuracy in ConvSNN.

We propose a novel data flow for the PIM-based processing of the TIFM. We read an input feature map (IFM) from the TIFM tensor, process the IFM using PIM, and generate the *V*_mem_ for output neurons. The sequential processing of an IFM overall spike cycles eliminates repeated reading of TIFM from DRAM and on-chip storage of *TV*_mem_. However, the sequential processing of IFMs introduces a bias in the STDP learning as IFMs processed earlier more strongly influence filter weights than the ones processed later. We propose a central STDP controller to ensure each filter is updated based on the IFM that results in the maximum *V*_mem_ of the firing neuron, rather than the IFMs that were processed earlier in sequence. In summary, our approach minimizes the data movement during inference, while ensuring the accuracy of the STDP learning process.

The accuracy of the accelerator is estimated considering MNIST, CIFAR-10, and CIFAR-100 datasets. With CIFAR-10 dataset, the accuracy with the weights trained by the standard STDP model is 67.88%. When we apply our modified STDP model, the accuracy is 66.25%, which is 1.63% lower than standard STDP model-based result. The experiment result demonstrates that on-chip and on-line STDP learning can be achieved with insignificant accuracy loss. The average power efficiencies of 18.69 TOPS/W and 7.25 TOPS/W are observed for inference and learning, respectively.

Along with a fully-STDP trained ConvSNN, the proposed architecture is also used to accelerate inference and on-line learning of a hybrid ConvSNN architecture that couples supervised (off-chip) trained and STDP (on-chip) learned layers. Previously, the concept of hybridization combining supervised training and STDP has been first introduced for a DNN (She et al., 2021). After that, Chakraborty et al. has shown the same concept of hybridization on SNN (Chakraborty et al., 2021). In this article, we show the hardware platform to accelerate the ConvSNN using the same concept of hybridization.

In addition to homogeneous networks, MONETAalso supports hybrid ConvSNN. Half of the layers can be on-line trained using the STDP algorithm and the other half of the layers are based on the externally programmed fixed weights. These fixed weights are off-chip trained by supervised learning. STDP uses unsupervised local learning to extract low-level features under spatial correlation. On the other hand, surrogate-gradient based backpropagation (BP) in ConvSNN enables global learning between low-level pixel-to-pixel interactions (Wu et al., 2018). It thus aids in high-level detection and classification similar to a SGD trained CNN model. By integrating global features using supervised training and local features using STDP learning, the hybrid network is also much more robust to local uncorrelated perturbations in pixels while extracting the correct feature representation from the overall image. Consequently, hybridization of surrogate-gradient and STDP enables robust image classification improving the accuracy of the baseline backpropagated ConvSNN model.

Based on the hybrid network simulation, we achieve 1.40% higher accuracy (77.83%) in MONETAthan the accuracy based on the supervised learning (76.43%) with the CIFAR-10 dataset. In addition, the average power efficiency for the hybrid on-line learning mode is 10.41 TOPS/W. This power efficiency is larger than on-line learning mode, but smaller than inference mode because half of the layer use inference mode and the other half of the layers use learning mode.

## 2. Background

### 2.1. ConvSNN and Unsupervised Learning Using STDP

**Figure 2:**
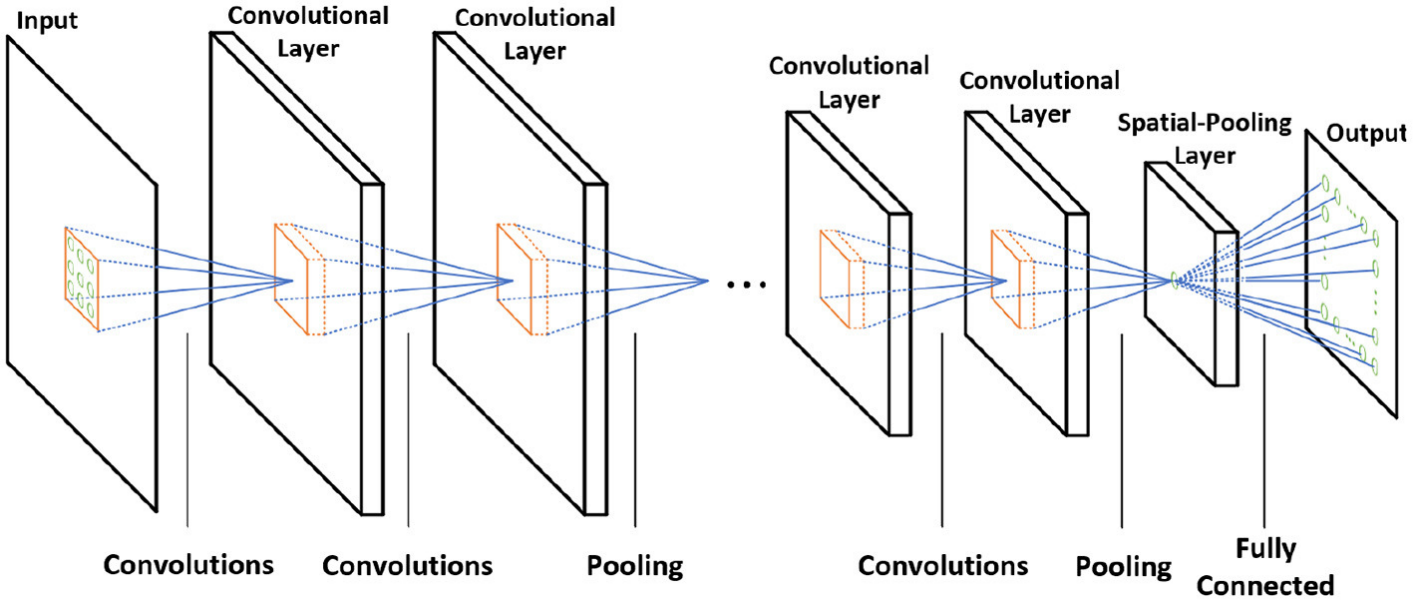
The architecture of the CNN.

**Figure 3:**
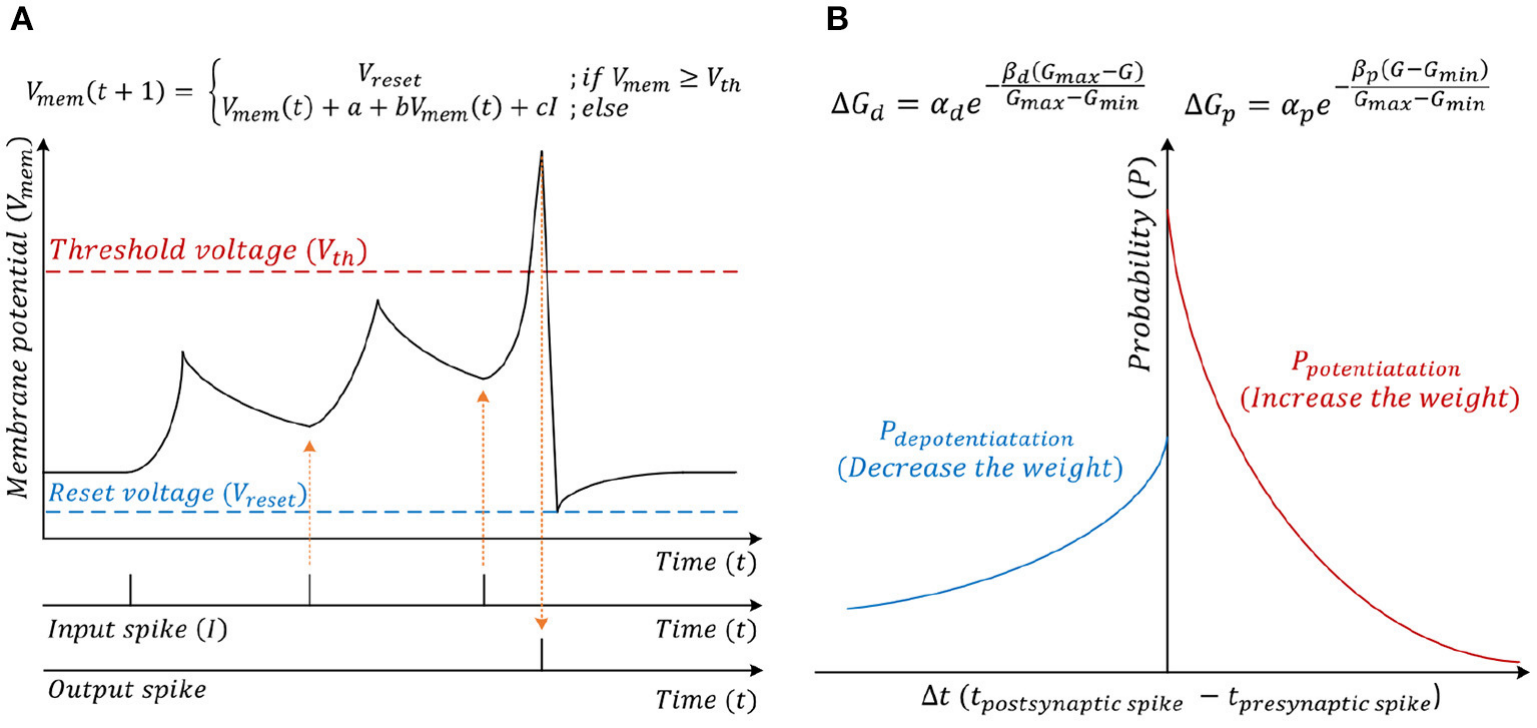
**(A)** Leaky integrate and fire (LIF) neuron computational model. **(B)** Stochastic spike-time-dependent-plasticity (STDP) model.

The spiking CNN uses the same structure as a traditional CNN (Figure 2). However, the input is a binary spike where the magnitude of the input. For example, the value of an image pixel is encoded in the frequency of the spikes. A spiking neuron computes the membrane potential *V*_mem_ using the spike levels multiplied by synaptic weights following the leaky integrate and fire (LIF) dynamics (Figure 3). An output spike is generated (neuron firing) when *V*_mem_ is higher than a threshold *V*_th_ and resets *V*_mem_ to *V*_reset_.

**Figure 4:**
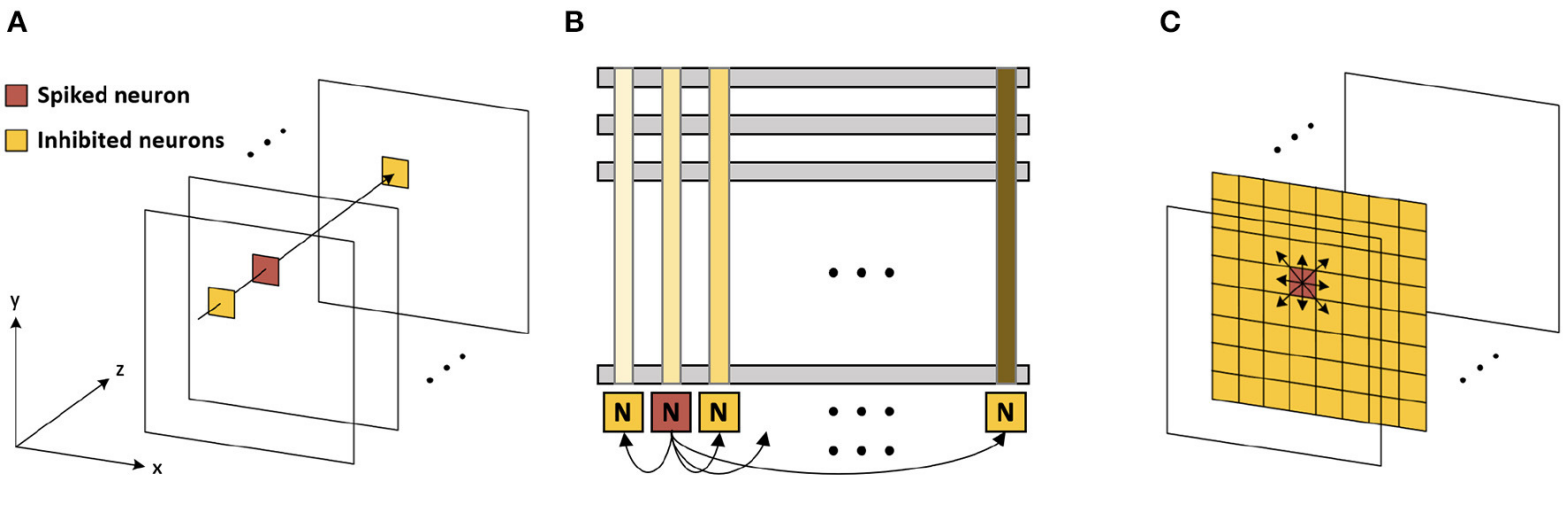
**(A)** Cross-depth inhibition **(B)** cross-depth inhibition on the memory array **(C)** lateral inhibition.

When the neuron fires (i.e., generates an output spike), the synaptic weights connected to the spiked neuron are updated following a stochastic STDP model (Figure 3B) (She et al., 2019). The firing of the neuron inhibits the firing of other neurons. There are two types of inhibitions, which are cross-depth inhibition and lateral inhibition. Figure 4A shows the cross-depth inhibition. In the case of the cross-depth inhibition, the firing of a neuron inhibits the firing of all other neurons located at the same (x, y) coordinates of all depths (across “z”-axis) in *TV*_mem_. The cross-depth inhibition can be easily implemented within the single PIM array and the neuron set (Figure 4B). In the case of lateral inhibition, the firing of the neuron inhibits all the neurons located at the same *z* coordinates (Figure 4C).

### 2.2. CNN Mapping for PIM Architecture

**Figure 5:**
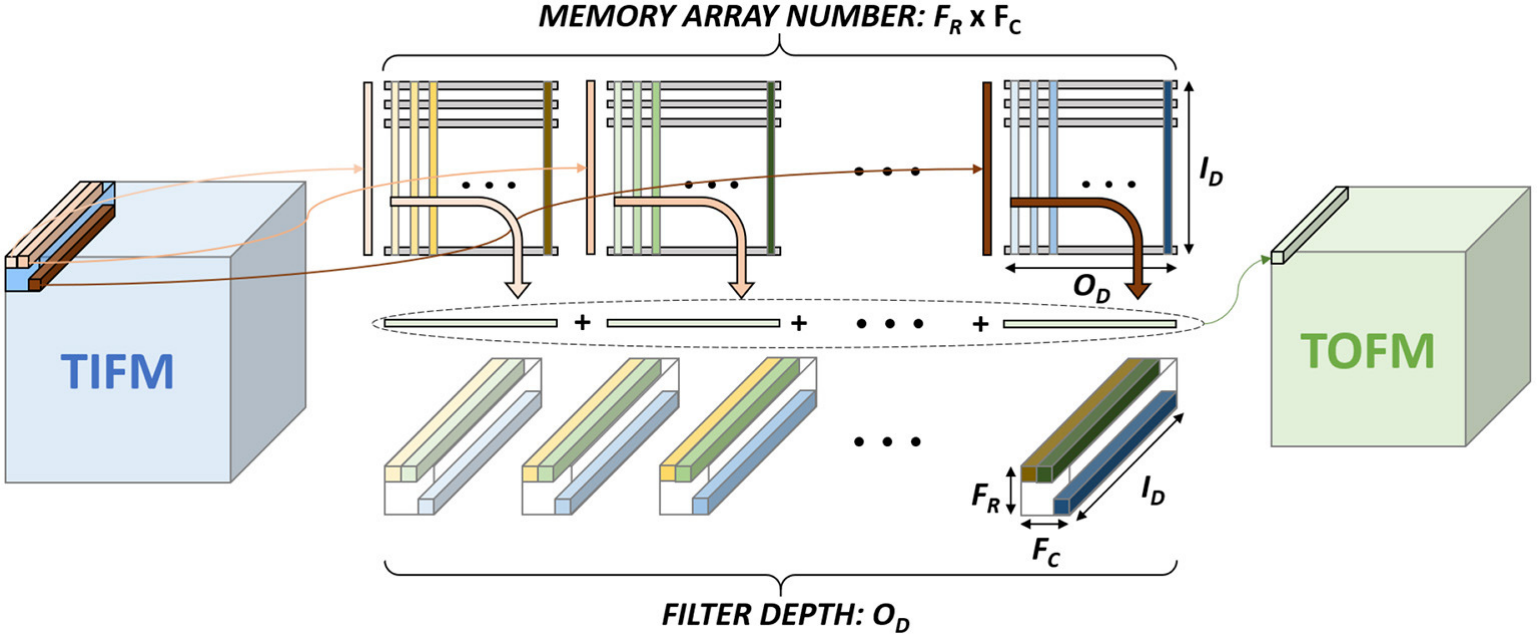
CNN mapping on the memory for processing-in-memory (PIM) architecture.

Figure 1A shows the basic terminologies for the CNN hardware (Chen et al., 2017). In the layer < n>, the size of TIFM is *I*_*R*_× *I*_*C*_× *I*_*D*_, the size of the filter is *F*_*R*_× *F*_*C*_× *I*_*D*_, and the size of total output feature maps (TOFM) is *O*_*R*_× *O*_*C*_× *O*_*D*_. The number of filters (depth of filters) are the same as the TOFM's depth (*O*_*D*_). The IFM, whose size is *F*_*R*_× *F*_*C*_× *I*_*D*_, is multiplied by each filter and generates the OFM, which size is 1 × 1 × 1. The stride is called as *S*. Figure 5 shows the CNN mapping method on the memory for the PIM architecture (Peng et al., 2021). Filter weights are divided by the x and y-axis, whose size is 1 × 1 × *I*_*D*_ and distributed on the different memory arrays. Also, each filter is placed on the different columns. To calculate the OFM, IFM is divided and sent to the memory sub-arrays. The multiplication between the synapse matrix and input vector is computed in each array, and outputs are summed to compute the OFM.

### 2.3. Prior SNN Accelerator Hardware

Various types of SNN based accelerators have been introduced in recent years. Buhler et al. (2017) made the analog neuron-based accelerator for the compact and energy-efficient design. However, they use the spiking locally competitive algorithm for an accelerator. Chen et al. (2019) showed the large-scale neuromorphic processor with 4,096-neuron and 1M-synapse. Their design uses binary activation, but the hardware is not optimized for the ConvSNN (Chen et al., 2019). Park et al. (2019) showed the ConvSNN based accelerator. However, they only used the stochastic gradient descent algorithm for the learning to improve the accuracy. In addition, the ConvSNN accelerator is introduced by Chuang et al. using a 2D systolic array with efficient data re-use, but their design does not include the on-chip training (Chuang et al., 2020).

Our design accelerates the ConvSNN using PIM architecture with on-chip STDP learning. The ConvSNN requires more complicated hardware design than multilayer perceptron-based SNN, but it has higher accuracy and lower memory usage for the weights on the complex image datasets such as CIFAR-10. The PIM architecture does not require the VMM calculation module, as we calculate the VMM in the SRAM array. In this sense, the PIM architecture can reduce the data transmission, as it does not require transmitting the weights to another module. In addition, the STDP learning rule benefits efficient learning for large-scale models or on-line learning as it enables unsupervised local learning. We propose a modified STDP algorithm to efficiently accelerate the PIM architecture.

### 2.4. Hybrid Spiking Neural Network

The SNN training methodologies can be broadly classified into three types: (1) conversion from artificial-to-spiking models (Diehl et al., 2015; Sengupta et al., 2019), (2) surrogate gradient descent based backpropagation with spikes (Lee et al., 2018; Wu et al., 2018; Neftci et al., 2019), and (3) unsupervised STDP based learning (Diehl and Cook, 2015; Srinivasan et al., 2018). Each technique has its own set of advantages and disadvantages. ANN-to-SNN conversion yields state-of-the-art accuracies, even for complex datasets like ImageNet (Deng et al., 2009) and can be used to convert complex architectures, like VGGNet (Simonyan and Zisserman, 2014), ResNet (He et al., 2016), RetinaNet (Miquel et al., 2021), the latency incurred to process the rate-coded image is very high (Pfeiffer and Pfeil, 2018; Lee et al., 2020). Surrogate gradient-based methods address the latency concerns but lag behind conversion in terms of accuracy for larger and complex tasks. The unsupervised STDP training also suffers from accuracy deficiencies. As pointed out by Panda et al. (2020), the accuracy loss due to vanishing spike propagation and input pixel-to-spike coding are innate properties of SNN design that can be addressed to a certain extent, but, cannot be completely eliminated. In order to achieve competitive accuracy as that of an ANN, previous works have taken a hybrid approach with a partly-artificial-and-partly-spiking neural architecture (Panda et al., 2020; She et al., 2020). As discussed by Ledinauskas et al. (2020), SNNs obtained by conversion must use only rate encoding, due to which the expressive capacity might be reduced. Another drawback of such conversion using rate-based encoding is that one needs to use forward propagation time steps in the order of thousands during the inference procedure for SNN. This drawback severely limits the computation speed and energy efficiency benefit of SNNs. Large spikes are necessary to reduce the uncertainty of spiking frequency values. Also, several ANN architectures are limited before conversion (e.g., batch normalization cannot be used) (Diehl et al., 2015; Sengupta et al., 2019). This limits ANN performance and the upper bound of SNN performance. Due to these limitations, we use a surrogate gradient-based method to train SNNs directly instead of converting ANN parameters to SNN.

**Figure 6:**
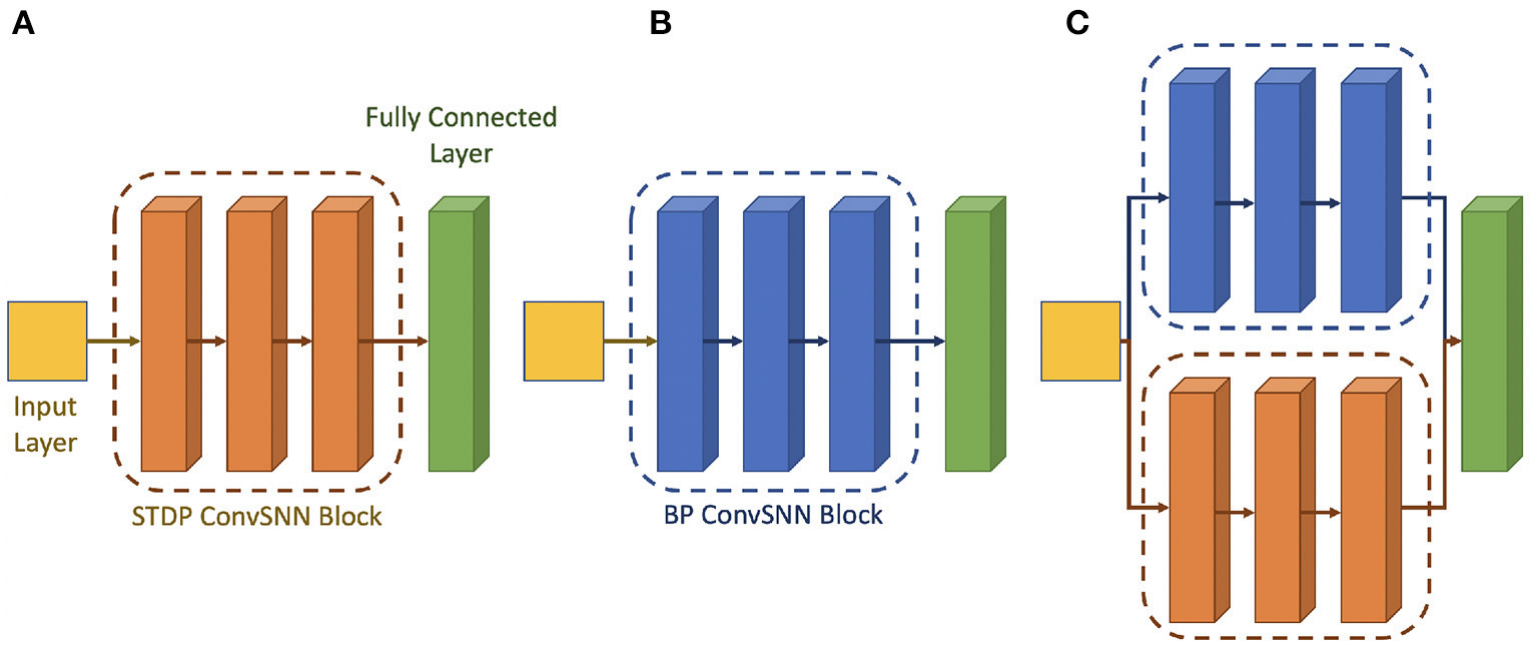
Architectural block diagram of the **(A)** STDP only network **(B)** backpropagation only network **(C)** hybrid Network.

Hence, following the work done by Chakraborty et al. (2021), we use a hybrid network consisting of surrogate-gradient based backpropagated ConvSNN modules along with the unsupervised STDP trained ConvSNN module. Figure 6 shows the architectural block diagrams of the different types of neural networks. Figures 6A,B show the homogeneous network architecture that uses STDP and the backpropagation, respectively. Figure 6C shows the hybrid network architecture whose weights are from the different training algorithms. The hybrid network consists of spiking layers placed in parallel to form different spiking convolution modules. The first spiking convolution module and half of the third spiking convolutional module (shown in blue in Figures 6A,C) are the backpropagated spiking modules. The second spiking convolutional module and the other half of the third spiking convolutional module (shown in orange in Figures 6B,C) are trained with the unsupervised STDP algorithm. The STDP-spiking convolution module is placed in parallel to the backpropagated module to enable robust extraction of local and low-level features. Further, to ensure that the low-level feature extraction also considers global learning, which is the hallmark of gradient back-propagation, several backpropagated ConvSNN layers of a similar size in parallel with the STDP ConvSNN module are used. The output feature map of the two parallel modules is maintained to have the same height and width and concatenated along the depth to be used as input tensor to the final ConvSNN layers. This ConvSNN module is responsible for higher level feature detection as well as the final classification. The main CNN module can be designed based on existing deep learning models. The concatenation of features from backpropagation-based ConvSNN and STDP-based ConvSNN modules help integrate global and local learning.

In addition, there exist other types of hybridization in the prior works. Lee et al. (2018) show the STDP-based unsupervised pre-training followed by supervised fine-tuning to improve the accuracy. Other works also show ANN-SNN hybridization that uses both ANN and SNN (Deng et al., 2020; Singh et al., 2020; Wang et al., 2021). On the other hand, hybridization in this article means, using only SNN with the different types of weight training algorithms (pre-trained backpropagation and STDP-based on-line leaning).

## 3. Hardware Architecture

**Figure 7:**
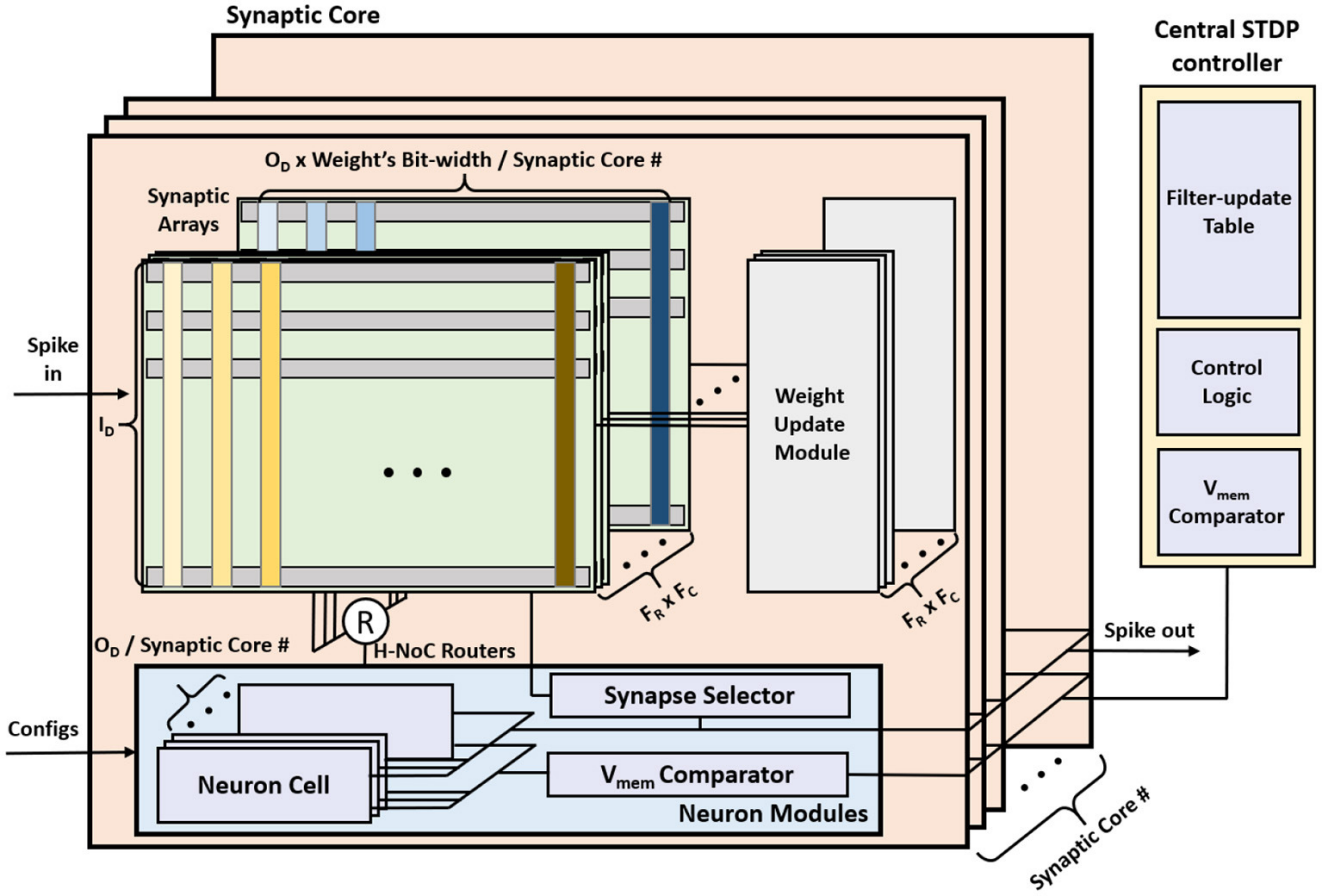
The MONETA system architecture overview.

The overall MONETAarchitecture consists of synaptic cores, neuron modules, and a central STDP controller (Figure 7). The synaptic core calculates the *V*_mem_ for each filter based on the IFMs and the weights. The synaptic array inside the synaptic core functions as a digital PIM core and calculates the vector matrix multiplication (VMM) of IFMs and synaptic weights. The results generated by the synaptic array are accumulated in the neuron module. The neuron module generates the output spikes based on the accumulated *V*_mem_ using the LIF model. The central STDP controller has a filter-update table and the training control module to control the synaptic core and the STDP-based weight update.

The STDP learning is performed using distributed weight update modules embedded in each synaptic core and a central STDP controller (Figure 7). The weight update module reads, computes the update, and writes back the synaptic weights using stochastic STDP (Kim et al., 2020). The central STDP controller manages the filter-update table and the learning process.

There are two phases in our design, the inference phase and weight update phase. In the inference mode, only the inference phase exists. In the learning mode, both the inference phase and the weight update phase exist. More precisely, during the inference phase in the learning mode, the central STDP controller collects the data in the filter update table while other modules do the same function with inference mode. After finishing the inference function for the scheduled cycles, MONETAstarts the weight update phase and updates the weights.

### 3.1. Proposed SNN Inference Methodology

An SNN receives the input as spikes. Based on each pixel's brightness, the range of the spike frequency is *f*_spike-min_~*f*_spike-max_. Assume, $T_{\text{spike}}\left(=\frac{1}{f_{\text{spike-max}}}\right)$ is a unit time-step, and *T*_total_ is a total exposure time for an input image. Therefore, ConvSNN (Figure 1B) receives all the IFMs, including the input image, for $N_{\text{spike-cycle}}\left(=\frac{T_{\text{total}}}{T_{\text{spike}}}\right)$ of spike cycles, computes the *V*_mem_ for all neurons, i.e., entire *TV*_mem_ tensor in each cycle based on the LIF neuron policy (Figure 3A). All the *V*_mem_ values in the *TV*_mem_ tensor that are higher than the threshold generate an output spike in the OFM.

#### 3.1.1. Sequential Processing of Spike Cycles

**Figure 8:**
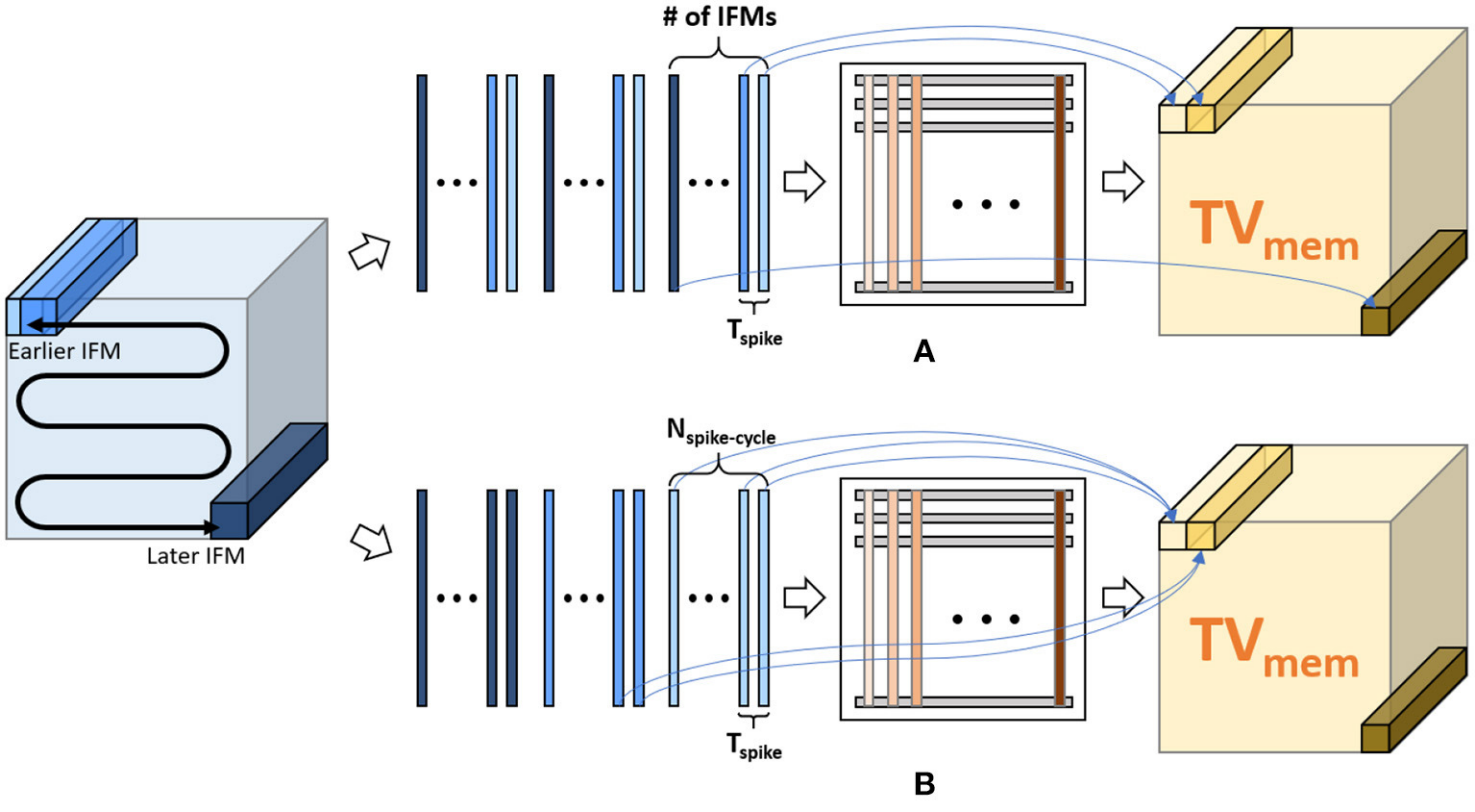
Inference approaches: **(A)** sequential processing of spike cycles: serially process all input feature maps (IFMs) in a total IFM (TIFM) for a given spike cycle and generate the entire *TV*_mem_. **(B)** sequential processing of IFMs: an IFM is serially processed for $N_{\text{spike-cycle}}\left(=\frac{T_{\text{total}}}{T_{\text{spike}}}\right)$ spike cycles followed by processing the next IFM.

Ideally, at spike cycle *i*, we need to generate a *TV*_mem_ tensor which is used along with the TIFM tensor to compute *TV*_mem_ for cycle *i*+1. Hence, in each spike cycle, we must compute all *V*_mem_ values in the *TV*_mem_ tensor by processing the all the IFMs in the TIFM tensor (Figure 8A). As all the IFMs are multiplied by same weight matrix, parallel processing of all the IFMs will require duplication of weight memory by $\frac{I_{\text{R}}}{\text{S}}\times \frac{I_{\text{C}}}{\text{S}}$ (where S is the stride), which is infeasible to store on-chip. Hence, we must process each IFMs serially in each accelerator clock cycle (*f*_CLK_ = 1 GHz in our design), as shown in Figure 8A). Hence, for each spike cycle, we can serially read all IFMs, multiply each IFM to all the filters in one accelerator clock *f*_CLK_, and serially compute all the elements of the *TV*_mem_ tensor. This is similar to operating a normal CNN. However, in ConvSNN, we must process the same TIFM tensor repeatedly for *N*_spike-cycle_ spike cycles in ConvSNN, such an approach requires either reading the same data (IFMs) from the off-chip memory repeatedly in every spike cycle resulting in a significant ($\frac{T_{\text{total}}}{T_{\text{unit}}}\times$) increase in data movement or store the TIFM tensor on-chip requiring large buffer. Moreover, we also need a global buffer to store the *TV*_mem_ tensor generated over the entire spike cycle. While processing spike cycle *i*+1, the global *TV*_mem_ buffer generated in the spike cycle *i* must be read by individual PIM blocks to generate the *TV*_mem_ tensor for the *i*+1 spike cycle. We will also need a global on-chip buffer of size *O*_R_× *O*_C_× *O*_D_ to store the *TV*_mem_ tensor increasing on-chip data movement between the PIM cores.

#### 3.1.2. Sequential Processing of IFMs

We propose to re-order the IFM processing as shown in Figure 8B. We first read one IFM and serially compute all *V*_mem_ values generated by that IFM over all *N*_spike-cycle_ spike cycles. Note, all these *V*_mem_ values can now be computed in *N*_spike-cycle_ of accelerator clock cycle. Moreover, as the IFM remains constant, the *V*_mem_ values for successive spike cycles can be locally accumulated within the PIM block eliminating the need for global *TV*_mem_ buffer and associated data movement. Moreover, serial processing of all spike cycles for a given IFM eliminates the need for repeated reading of the entire TIFM tensor thereby reducing off-chip data movement.

### 3.2. Hardware Support for Inference

#### 3.2.1. Synaptic Core

The synaptic core is used for distributed computation of *V*_mem_ and generates the output spike. The synaptic core uses synaptic arrays (weight storage), routers, and neuron modules for the inference. Since the weight matrix is distributed across multiple synaptic cores, each synaptic core has a subarray of dimension $I_{\text{D}}\times \frac{O_{\text{D}}\times \text{weight's bit-width}}{\text{\# of synaptic core}}$, and calculates the matrix multiplication results for $\frac{O_{\text{D}}}{\text{synaptic core \#}}$ filters.

#### 3.2.2. Synaptic Array

**Figure 9:**
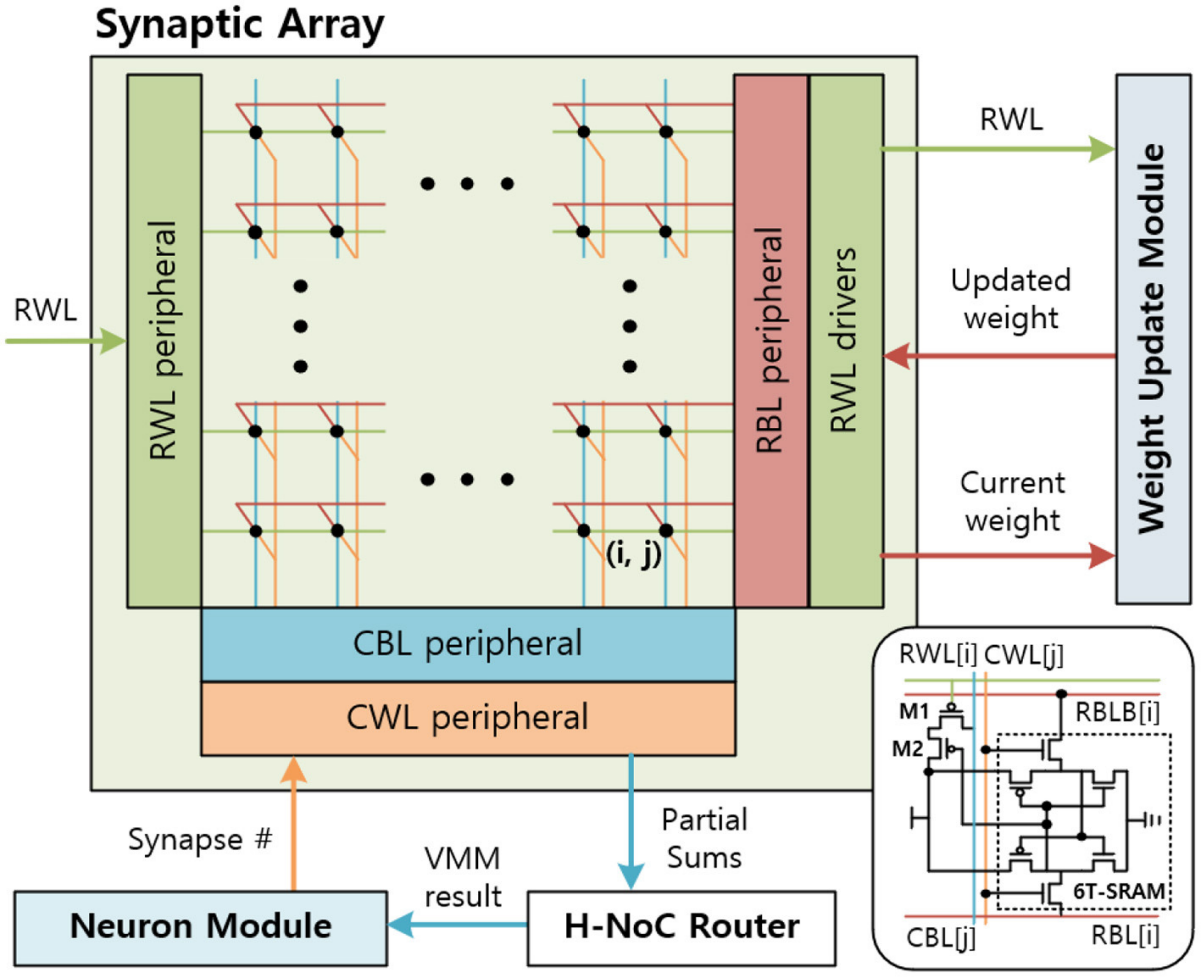
The synaptic array architecture.

The synaptic array multiplies the IFMs and synaptic weights to generate the partial sum of the VMM result. A sequential (row-by-row) read access-based PIM design is considered for synaptic arrays to multiply IFMs and weights. Then, the hierarchical network-on-chip (H-NoC) router adds partial sums and sends the VMM result to the neuron module. The synaptic array is implemented by SRAM array, peripherals, and drivers (Figure 9). Synaptic weights are 8 bits and consist of 8 consecutive SRAM cells in a row. The most left SRAM cell represents the sign bit.

The synaptic array receives the input spikes of the IFM on the row-wise word-line (RWL) port. The input spikes are sent in row-by-row order, so the RWL peripheral uses a counter-based decoder to send input spikes to an 8T-SRAM array sequentially. When the result of sense amplifier for CBL is 1, the CBL peripheral sends the partial sum, 1, to the H-NoC router and pre-discharges the CBL. The H-NoC connects the synaptic arrays, accumulates the partial sums, and sends the VMM result to the neuron module (Long et al., 2019).

#### 3.2.3. Neuron Module

**Figure 10:**
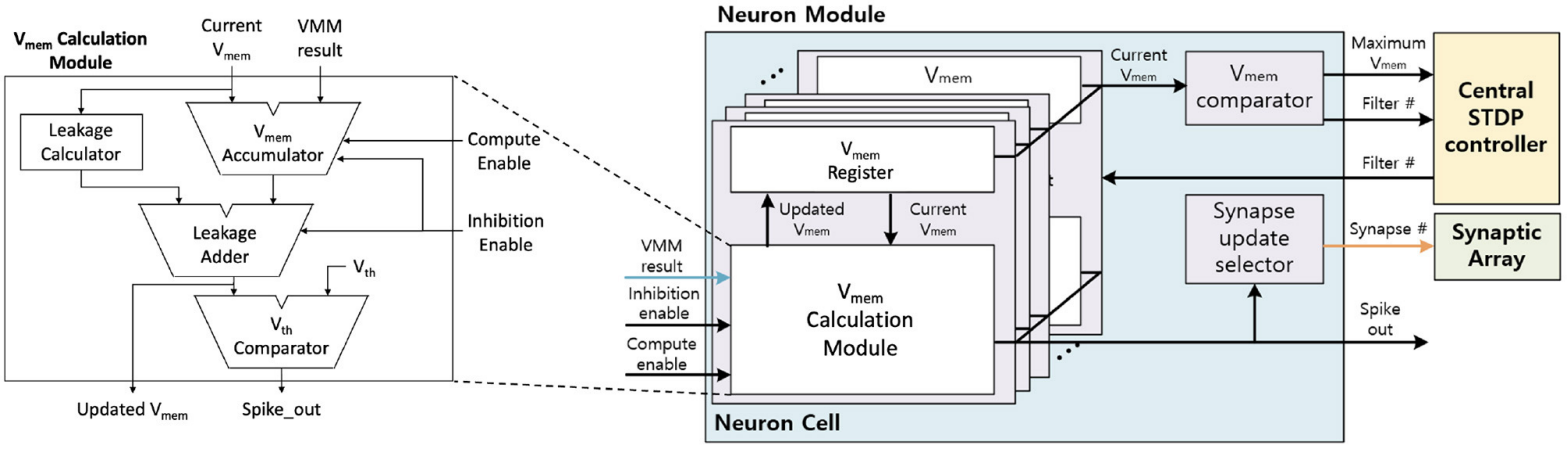
The LIF Neuron Module architecture.

The neuron module receives the VMM result from the synaptic arrays, calculates the *V*_mem_, and generates the output spike. Figure 10 shows the neuron module architecture. The neuron module consists of $\frac{O_{\text{D}}}{\text{synapse core}\#}$ neuron cells, *V*_mem_ comparator, and synapse update selector. The neuron cell updates the *V*_mem_ based on the LIF neuron dynamics and generates the output spike when the *V*_mem_ over the *V*_th_. *V*_mem_ comparator and Synapse update selector are disabled during inference. These modules are discussed in section 3.3

To generate the output spike, the neuron cell receives the VMM result and updates the *V*_mem_ based on the LIF neuron dynamics. Inside the *V*_mem_ calculation module (Figure 10), VMM result and the current *V*_mem_ are added when the compute enable signal is enabled. This *V*_mem_ accumulation takes *I*_*D*_ cycles, as the whole VMM computation requires *I*_*D*_ cycles with row-by-row access on synaptic array. Then, the leakage calculator calculates the leakage based on the current *V*_mem_ and subtracts the leakage to the result of the *V*_mem_ accumulator to generate the updated *V*_mem_. In the end, if the updated *V*_mem_ is larger than *V*_th_, the neuron cell will generate the output spike and reset *V*_mem_ as 0. Updated *V*_mem_ is stored in the *V*_mem_ register inside the neuron cell to be used in the next time step.

### 3.3. Proposed PIM-Friendly STDP Learning Methodology

We argue that the proposed approach of sequential processing of IFMs can lead to bias in STDP learning. In ConvSNN with cross-depth inhibition, each depth controls the weight update for a filter tensor. Consider a neuron at location *x*_*k*_, *y*_*k*_, *z*_*k*_ fires, then it will inhibit the firing of all other neurons across the depth, i.e., all neurons at *x*_*k*_, *y*_*k*_ but all locations across the *z*-axis. In an ideal case, *V*_mem_ values of all the neurons in the same depth of the *TV*_mem_ tensor are calculated simultaneously. Hence, for a given depth, the neuron with the maximum *V*_mem_ considering the entire TIFM will fire and control the weight update process for the associated filter. However, when IFMs are processed sequentially, the STDP based updates of filter weights are controlled by the order in which IFMs are processed. For example, considering the order shown in Figure 8B, the IFM in the earliest position (top-left segment in the TIFM tensor) can cause firing at a given depth change with the associated filter weights. The *V*_mem_ computation for the later IFMs will be performed with the already changed filter weights and hence will have less impact on overall learning. This leads to undesired sequential bias in the STDP learning.

**Figure 11:**
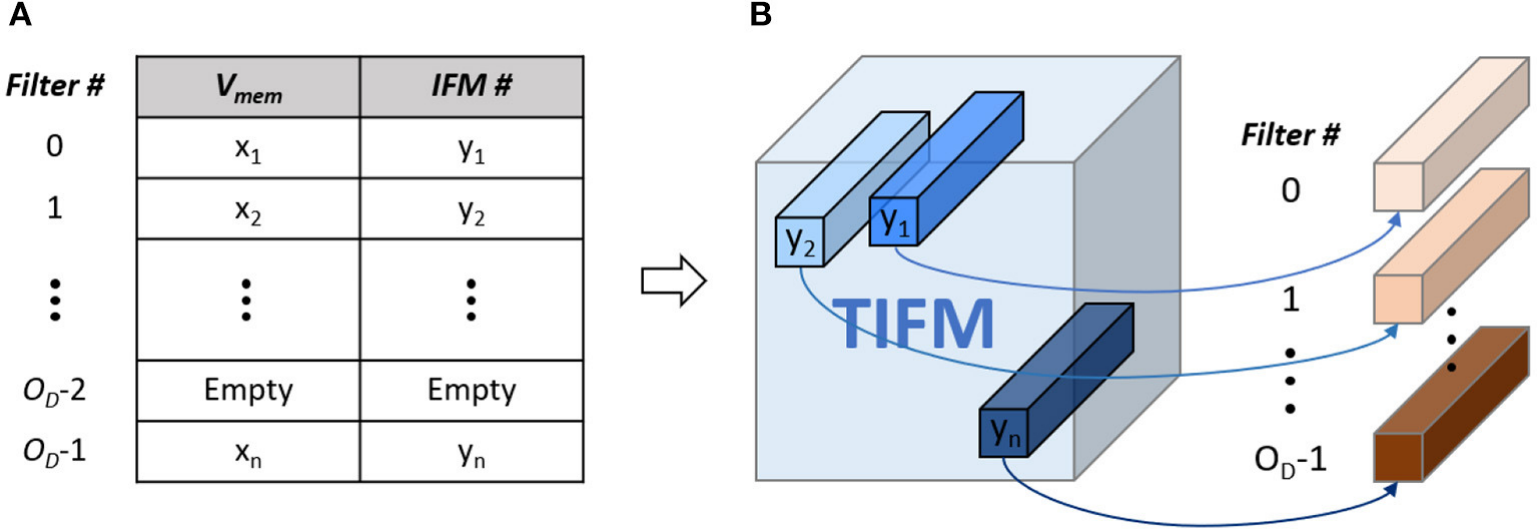
**(A)** Filter-update table **(B)** relation between IFM number and target filter based on the filter-update table.

We address this problem by ensuring that at a particular depth the neuron which has the maximum *V*_mem_ considering all IFMs control STDP-based update of the corresponding filter weight (shown in Figure 1B). This is achieved by maintaining a central filter-update table where for each filter we store a running value of the maximum *V*_mem_ and corresponding IFM number (Figure 11A). While processing the “*i*th” IFM over all spike cycles, we compute *V*_mem_, fire a neuron (as required), reset *V*_mem_ for all other cross-depth neurons but do not initiate weight update. Instead, we estimate the *V*_mem_ values for all the neurons at all depths due to the “*i*th” IFM. If at a given depth, the *V*_mem_ generated by “*i*th” IFM is higher than the maximum *V*_mem_ value stored in the table for the corresponding filter, we update the central table to indicate “*i*th” IFM results in the maximum *V*_mem_ for this filter. The table generation is finished after processing all the IFMs. Once completed, we show all the IFMs one more time and update the filter weights based on the filter-update table (Figure 11B). The overhead is cost of processing TIFMs two times, one for generating the filter-update table and the second for updating the weights (Figure 3B). Therefore, our PIM-friendly STDP learning can train the weights based on the STDP algorithm without considerable IFM movements and the bias occurring from the sequential IFM processing.

### 3.4. Hardware Support for Learning

#### 3.4.1. Synaptic Core

In the learning mode, the synaptic core uses synaptic arrays (weights storage), routers, neuron modules, and weight update modules. The weight update module is power gated in the inference mode but is used in the learning mode. Synaptic core and neuron modules calculate the *V*_mem_ and generate the output spike. When the output spike is generated, weights are updated with the control from the central STDP controller.

#### 3.4.2. Synaptic Array

The SRAM array in the synaptic arrays is implemented by an 8T-SRAM array. 8T-SRAM allows transposable read and write thereby allowing parallelism in weight update (Seo et al., 2011; Kim et al., 2020). Figure 9 shows the synaptic array and its connections with other modules. The 8T-SRAM includes the 6T-SRAM, the PMOS M1, and the PMOS M2. The 6T-SRAM stores the synapse weight, and the PMOS M1 and the PMOS M2 connect RWL, synapse weight, and column-wise bitline (CBL) for the matrix multiplication. When the RWL sends the spike and the synapse weight bit is 1, CBL is charged.

During the weight update phase, the syna does the same inference function until the neuron module generates the output spike. When the output spike is generated, the synaptic array receives the synapse number from the neuron module and decodes it to generate the column-wise wordline (CWLs) to read the SRAM data stored in the 6T-SRAM cell, included in the 8T-SRAM. Total 8 CWLs are generated sequentially for each clock to read the 8-bit synapse weight information. The CWL is connected to the 6T-SRAM cells' CWL vertically and reads the data by RBL and RBLB horizontally. The RBL peripheral reads the synapse weight data for each clock and sends it to the weight update module. After the weight update module calculates the synaptic weights, RBL peripheral receives the updated synapse weights and writes them back to the 6T-SRAM cells.

#### 3.4.3. Neuron Module

In the learning mode, *V*_mem_ comparator and the synapse update selector are additionally used. During the inference phase in the learning mode, the neuron module compares the *V*_mem_ at the *V*_mem_ comparator and sends the maximum *V*_mem_ and the filter number to the central STDP controller for each IFM. In the weight update phase, the neuron module receives the active filter number from the central STDP controller, and only the selected filter calculates the *V*_mem_. The selected filter calculates the *V*_mem_ in the neuron cell and generates the output spike when the *V*_mem_ is over the threshold voltage. When the neuron cell generates the output spike, the neuron cell resets the *V*_mem_ to 0 and holds the *V*_mem_ calculation while the synapse array updates the weight. The synapse update selector receives the output spike, generates the synapse number, and sends this number to the synapse array.

#### 3.4.4. Weight Update Module

**Figure 12:**
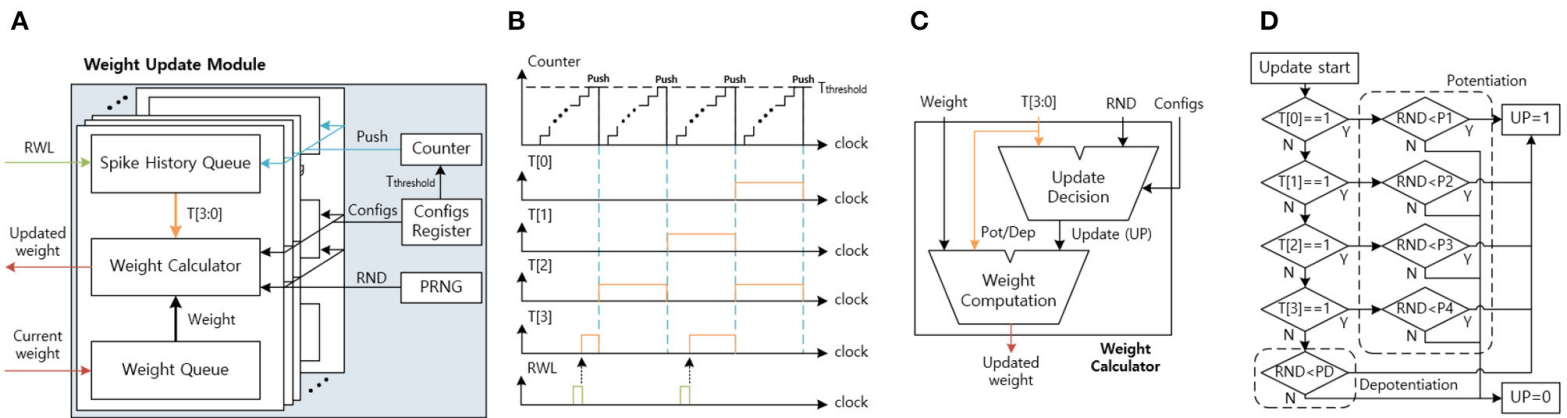
**(A)** The weight update module architecture. **(B)** The spike history queue function graph. **(C)** The weight calculator architecture. **(D)** The update decision module's state machine for stochastic STDP.

Figure 12A shows the architecture of the weight update module. The weight update module calculates the updated weights based on the current weights and the timing information using the stochastic STDP rule. The timing information is used to check the probability of potentiation or depotentiation (Figure 3B). The configuration register (configs register) stores the programmable configurations for the timing queue control and the stochastic STDP rule. The pseudo-random number generator (PRNG) generates the random number (RND), which decides whether to update or not to update weights and is implemented by linear-feedback shift registers (LFSRs). The counter is used to push the spike history queue.

The spike history queue receives the RWL, delivered from the synaptic array, and stores the input spike history in the spike history queue (Figure 12B). The T[3] is connected to the RWL and is set to 1 when the RWL is 1. When the counter reaches the threshold time (T_threshold_), reset the T[3] to 0 and push the queue from T[3:1] to T[2:0]. As a result, the spike history queue stores the input spike history, and by changing the T_threshold_ in the configs register, we can control the spike history period.

The weight queue receives the current weight from the synaptic array one bit per clock until it receives all 8-bit of the synapse weight. After the weight queue receives the current weight, the weight calculator calculates the updated weight based on the spike history and the current weight (Figure 12C). The weight update calculator includes the update decision module and the weight computation module. The update decision module determines whether to update the weight or not according to the stochastic STDP rule. When the update (UP) signal is 1 and the T[3:0] is all 0, the weight computation module decreases the weight. When the UP signal is 1 and the T[3:0] has at least 1, the weight computation module increases the weight. At the end, when the UP signal is 0, the weight computation module does not change the weight. After the updated weight calculation, the weight update module sends the updated weight one bit per clock to the synaptic array.

As shown in Figure 12D, the update decision module's state-machine describes the stochastic STDP. The update decision module receives the input spike history, the RND, and the configurations (configs). The configurations include the potentiation thresholds (P1, P2, P3, and P4) and the depotentiation threshold (PD). The spike history determines which potentiation/depotentiation threshold will be used. The UP is set to 1 when the RND is smaller than the selected threshold. When the RND is equal to or larger than the threshold, UP is set to 0.

#### 3.4.5. Central STDP Controller

**Figure 13:**
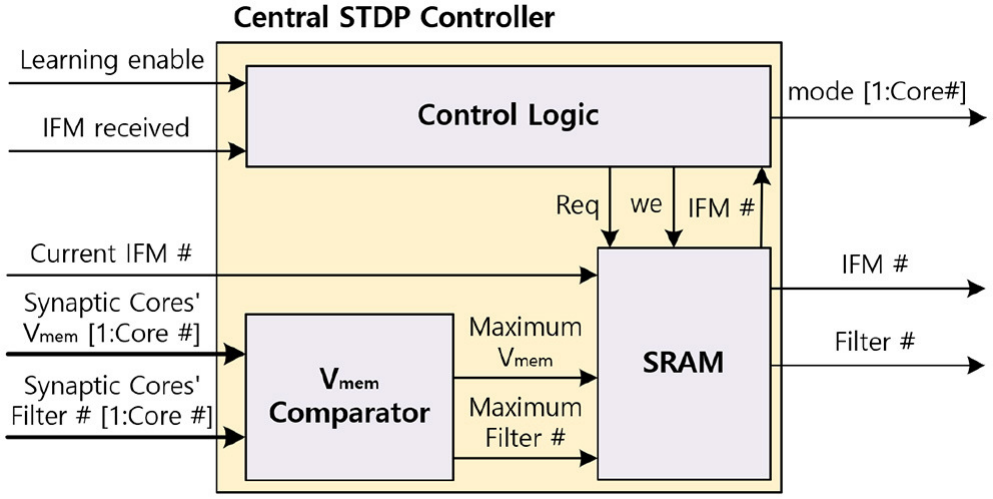
The central STDP controller architecture.

The central STDP controller includes SRAM which stores the filter update table, the *V*_mem_ comparator to find the maximum *V*_mem_ of MONETA, and the control logic to control MONETA. The central STDP controller controls the design and determines the weights to update during the learning mode. Figure 13 shows the architecture of the central STDP controller.

During the inference phase in the learning mode, MONETAfills the filter update table. To generate the data for the filter update table, the synaptic core receives the IFM and calculates the membrane potential. During this process, the neuron modules calculate the *V*_mem_ and send the maximum *V*_mem_ and the corresponding filter number to the central STDP controller. The central STDP controller compares the current IFM's maximum *V*_mem_ and the previous IFM's *V*_mem_ which is stored in the filter update table. If the current IFM's maximum *V*_mem_ is larger, the filter update table will store the current IFM and the new maximum *V*_mem_ in the filter update table. When the weight update phase starts, the central STDP controller reads the IFM for the maximum *V*_mem_ from off-chip memory for each filter. The IFM is applied to the target synaptic core to re-compute the corresponding *V*_mem_, generate the output spikes, and update the weights using the weight update modules. Once all the filters are updated for a TIFM, the filter update table is reset.

**Figure 14:**
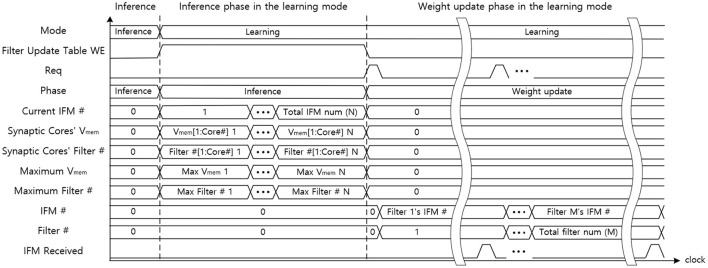
The data flow graph of the central STDP controller.

Figure 14 indicates the data flow timing diagram of the central STDP controller. The central STDP controller receives the mode signal from the user to determine the mode of the synaptic core. The central STDP controller controls the function of synaptic cores by sending the phase and mode signals to the synaptic cores. During the inference phase in the learning mode, the central STDP controller receives the maximum *V*_mem_ of the synaptic cores, filter number from the synaptic cores, and the current IFM number from the off-chip memory to generate the filter update table. In the weight update phase, the control logic request the IFM number to the filter update table in the SRAM with the request (Req) signal. The filter update table sends the updating target IFM number to the off-chip memory and the updating target filter number to the updating target synaptic core. The received IFM signal transmitted from the off-chip memory is used to determine whether the IFM is delivered from off-chip memory during the weight update phase. This is because the central STDP controller sends non-sequential IFM requests to the off-chip memory, so the MONETAneeds to be in the idle state until the IFM is transmitted. The control logic also receives the filter number. After that, the control logic generates the mode signal to the target synaptic core. Only the updating target synaptic core is enabled to update weights by mode signal and other synaptic cores are in the idle state as they do not need to update the synapse weights. This process is continued for all the filters in the filter update table.

### 3.5. Hybrid Network With Coupled Supervised and Unsupervised Learning

As we discussed in section 2.4, hybrid networks can help us improve the accuracy of the STDP network. Thus, in this article, we used a hybrid supervised-unsupervised learning methodology similar to the works done by Chakraborty et al. (2021). Supervised learning is the surrogate gradient-based training of the SNNs (Wu et al., 2018; Neftci et al., 2019). The supervised learning-based weights are trained at the off-chip, then are loaded on the synaptic array. These supervised learning-based layers are set as the inference mode (marked blue in Figure 6C). The unsupervised learning algorithm is our modified STDP-based learning. These layers are set as the learning mode and the weights are trained on-chip. Therefore, because our design supports on-line learning, half convolutional layers have supervised learning-based fixed weights and the other half of convolutional layers have unsupervised on-line learning-based flexible weights (marked orange in Figure 6C).

## 4. Simulation Results

### 4.1. Configurations of Simulated ConvSNN

As discussed before, we use both homogeneous and hybrid networks. Hybrid networks with supervised training can help us improve the accuracy of the STDP network. Thus, we simulate the hybrid supervised-unsupervised learning methodology similar to the works done by Chakraborty et al. (2021).

**Configurations:** We define the type of networks to compare our hybrid spiking neural network for image classification on the MNIST and CIFAR-10 dataset as follows:

- **Standard STDP model (Type 1):** we use the 4-layer ConvSNN model trained using the standard STDP model (Bi and Poo, 1998)
- **PIM-friendly STDP model (Type 2):** we use the 4-layer ConvSNN model and train it using the modified STDP rule explained in section 3.3
- **Fully Backpropagated ConvSNN model (Type 3):** for this model, we use another backpropagated ConvSNN block instead of the STDP ConvSNN block (orange block in Figure 6). This makes the entire model to be trained with a surrogate gradient without any unsupervised STDP block.
- **Hybrid model with standard STDP model (Type 4):** for this model, we use the hybrid network as shown in Figure 6C. However, we use the standard STDP learning rule for the STDP ConvSNN block (orange block).
- **Hybrid model with PIM-friendly STDP model (Type 5):** this is the proposed model using hybridization of STDP-based ConvSNN and backpropagated-based ConvSNN blocks. The STDP learning rule used to train the STDP block is the modified STDP rule as discussed in section 3.3

**Types 1–3** are based on the homogeneous network architecture. The weights of architectures in **Types 1–3** are trained by single training algorithm. **Types 4-5** are based on the hybrid network architecture we discussed in section 2.4. Half of the weights in **Types 4-5** are trained by backpropagation algorithm and the other half of the weights are trained by different STDP algorithms for each network type.

### 4.2. Hardware Architectures for Simulation

Table 1 shows the simulated ConvSNN network architecture with four convolutional (CONV) and one fully-connected (FC) layer. We use 8-bit precision for the weights and 4-bit precision for the input spikes. The total on-chip memory used for synaptic cores is determined by the filter size of the **CONV4** layer in the homogeneous network architecture. Therefore, we need two MONETAchips for the **CONV4** layer in the hybrid network. We divide the total capacity into 8 synaptic cores where each core has nine 128 × 128 synaptic arrays. We consider on-chip STDP is performed using a layer-by-layer fashion because OFMs for one layer are used to train the next layer. Note the memory capacity is sufficient to simultaneously map **CONV1**, **CONV2**, and **CONV3** on the chip during inference. We consider that the **FC** layer exists off-chip and connected with MONETA.

**Table 1:** Architecture and parameters of the tested convolutional neural network within spiking neural network (ConvSNN) networks.

**Layers**	**Homogeneous network architecture**			**Hybrid network architecture**					

				**BP ConvSNN block**			**STDP ConvSNN block**		
	***F***_**R**_	***F***_**C**_	***F***_**D**_	***F***_**R**_	***F***_**C**_	***F***_**D**_	***F***_**R**_	***F***_**C**_	***F***_**D**_
CONV1	3	3	64	3	3	64	3	3	64
CONV2	3	3	64	3	3	64	3	3	64
CONV3	3	3	128	3	3	128	3	3	128
CONV4	3	3	128	3	3	128	3	3	128
FC	***F***_**R**_	***F***_**C**_	***F***_**D**_	***F***_**R**_		***F***_**C**_		***F***_**D**_	
	1	1	512	1		1		1,024	

**Figure 15:**
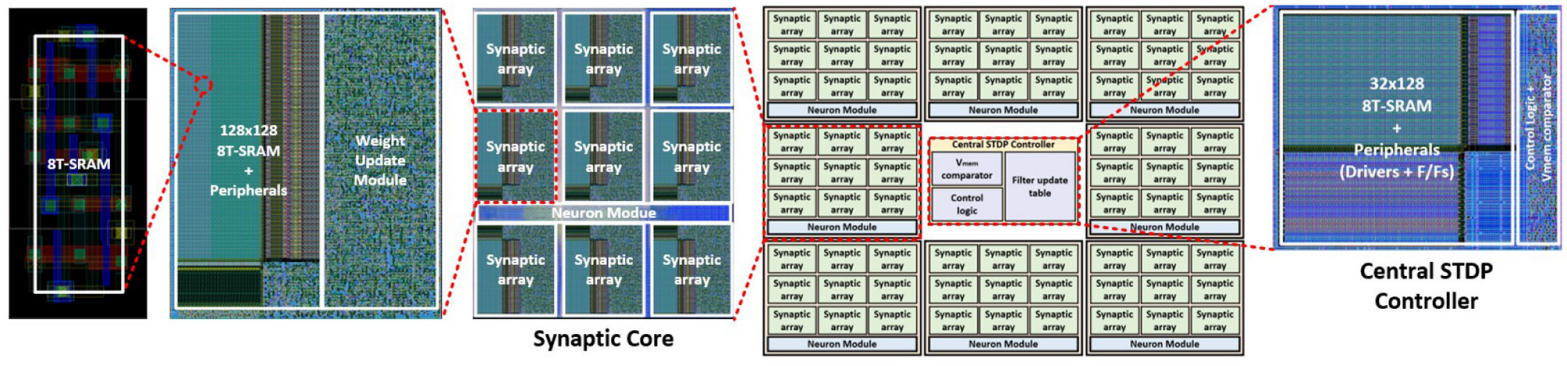
The overview of the physical design of MONETA.

The hardware architecture of MONETAwith 8 synaptic cores and one central STDP controller is implemented in 65 nm CMOS (Figure 15). We used the Virtuoso for the full-custom layout of 128 × 128 SRAM sub-arrays and Innovus for the auto place and route (PNR) of other logic blocks. Each synapse core and the central STDP controller have 1.394 and 0.025 *mm*^2^ areas. The throughput and power of the design are estimated from the layout and after parasitic extraction.

### 4.3. Accuracy Analysis

The ConvSNN shown in Table 1 is simulated using ParallelSpikeSim, an open source GPU accelerated SNN simulator (She et al., 2019). The MNIST and CIFAR-10 datasets are used for accuracy evaluation. All synapses are designed with 8-bit weights. The unsupervised-learning based CONV layers are trained with STDP for unsupervised clustering of inputs. The supervised-learning based CONV layers are trained with the BPTT algorithm. The final FC layer is trained using Stochastic Gradient Descent (SGD) to label the clusters with appropriate classes. Input spike frequency is converted from image pixels intensity to the range of 10–50 Hz. We assumed $\frac{T_{\text{total}}}{T_{\text{unit}}}=100$, i.e., each image is shown to the network for 100 time steps. Each layer learns the entire training set for 5 epochs.

Table 2 shows the accuracies of each ConvSNN configuration. **Type 2** is based on the data flow of MONETA (Figures 8B, 11). When we compare the accuracies with CIFAR-10, the accuracy of **Type 2** shows only 1.63 (%) lower accuracy than a fully parallel (as shown in Figure 8A) software implementation of the network using 8-bit precision (**Type 1**). As mentioned before, the fully parallel implementation incurs $\frac{T_{\text{total}}}{T_{\text{unit}}}\times$ (=100 × ) higher data movement than our design. The accuracy of the ConvSNN accelerated using MONETA (**Type 2**) is 10.18% lower than a spiking neural network of the same layer configurations trained using backpropagation (**Type 3**). In the end, the accuracy of the hybrid network shows improved accuracy than supervised learning. The result of the hybrid network using backpropagated-based ConvSNN and the PIM-friendly STDP learning (**Type 5**) shows 1.4% higher accuracy than the fully backpropagated ConvSNN model (**Type 3**). This accuracy from **Type 5** is only 1.11% lower than the hybrid network applying the standard STDP model (**Type 4**).

**Table 2:** Simulated network types and the results.

**Type**	**Learning algorithm**	**Required** **parameters** **(Kb)**	**Inference** **throughput** **(TOPS)**	**Inference** **Energy** **efficiency** **(TOPS/W)**	**On-line** **learning** **throughput** **(TOPS)**	**Learning** **energy** **efficiency** **(TOPS/W)**	**Accuracy** **(CIFAR-100)**	**Accuracy** **(CIFAR-10)**	**Accuracy** **(MNIST)**
**1**	Standard STDP	1,152	2.304	18.69	N/A	N/A	54.25	67.88	90.89
**2**	PIM-friendly STDP				2.2	7.25	52.19	66.25	90.13
**3**	Backpropagation (BP)				N/A	N/A	62.12	76.43	92.55
**4**	BP + Standard STDP	2,304	4.608		N/A	N/A	63.86	78.94	93.16
**5**	BP + PIM-friendly STDP				4.4	10.41	62.31	77.83	92.07

We note that the accuracy of backpropagation-based trained SNN demonstrated in this article is lower than the state-of-the-art, for example, 99.59% accuracy was observed on MNIST dataset (Lee et al., 2020). This is primarily because, we have reduced the simulation time necessary for training the network with back propagation. For example, instead of 100 epochs of training as performed in the Lee et al. (2020) we only trained the network for 20 epochs. Further, we did not apply pre-processing and the fine- tuning methodologies that are normally applied in BP SOTA, as these techniques are applied off-chip and are not related to the PIM-based hardware implementation of ConvSNN.

### 4.4. Throughput Analysis

Table 2 shows the peak throughput of MONETAestimated as Tera Operation per Second (TOPS). The throughput for each synaptic array is determined by the number of parallel multiplies (# of weights stored in a row) and accumulate. The total throughput of all synaptic arrays is given by # of weights × # of synaptic arrays × frequency. H-NoC sums partial outputs from synaptic arrays resulting in a throughput of # of weights × # of synaptic array × frequency. The neuron modules compute membrane potential neurons at a throughput of # of weights × frequency. The total throughput is obtained considering the parallel operation of all synaptic cores. Our design has # of weights per word line = 16, # of synaptic arrays = 9, # of synaptic cores = 8, and frequency = 1GHz. Hence, the total throughput of one MONETAchip is 2.304 TOPS in the inference mode. On the other hand, in the case of the on-line learning mode, the throughput is reduced based on the time used for the training. Because the weight update takes 17 cycles, the throughput becomes 2.304$\times \frac{1}{1+17\times \text{(output spike rate)}}$. For example, learning mode throughput in **CONV4** layer is 2.2 TOPS with an output spike rate of 0.0028.

In MONETA, each time-step is represented as 1 clock cycle (1 GHz), and 100 time-steps are used for each image. The total clock cycles required to operate on one image in each layer is $100\times \frac{I_{\text{R}}}{\text{S}}\times \frac{I_{\text{C}}}{\text{S}}\times I_{\text{D}}$ (S is a stride), where *I*_D_ represents a number of rows in the synaptic array. We compute the image processing rate (fps) of **CONV1-4** is 13.02, 2.44, 9.77, and 19.53 K, respectively, at 1 GHz.

### 4.5. Area and Power Analysis

The power of the MONETAdesign is 123.28 mW, 303.52 mW for the inference mode and the learning mode, respectively, at 1 GHz with 1 V supply on the one chip. This power is calculated based on **CONV4** which is the maximum power of MONETA. In addition, the power is computed considering **CONV4**'s input and output spike activity ratio (0.0092 and 0.0028). Note, **CONV4** of the hybrid network requires two MONETAchips because of its parameters, so the total power is two times the homogeneous networks (246.56 mW and 607.04 mW for the inference mode and the learning mode, respectively).

**Figure 16:**
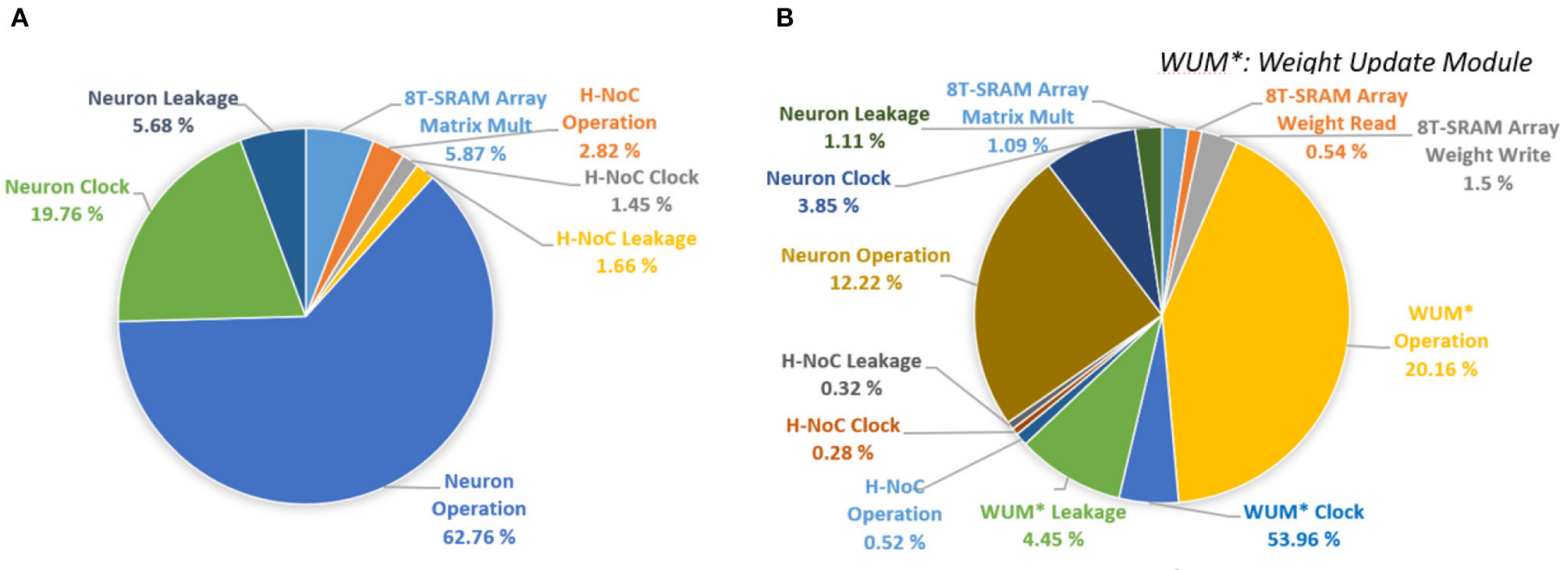
Power breakdown of MONETA. **(A)** inference mode and **(B)** training mode.

Figure 16A shows the power breakdown of the synaptic core's inference mode. The weight update module is idle during the inference mode by clock gating. The 8T-SRAM array consumes the 21.86 pJ for matrix multiplication calculation, 4.48 pJ for transposable weight read, and 12.34 pJ for transposable weight write. The SRAM-based computation naturally transforms sparsity in neuron firing (i.e., zero values in IFM) to power saving during inference. If an input spike is absent in a cycle, the SRAM power for that cycle is zero as word lines are not activated. Moreover, as we use single ended sensing in 8T-SRAM, there is no bit-line discharge when the values of the corresponding bit are “0.” Hence, the SRAM contributes very little power to the overall operation. The power is dominated by the *V*_mem_ calculation in the neuron module. This is because there exists an inherent leakage component in the membrane potential computation (*a*+*bV*_mem_ in LIF dynamics in Figure 3) that causes the membrane potential to reduce when there are no input spikes. Hence, the neuron module needs to perform the leakage computation in each clock. However, the power in the synaptic array and the H-NoC reduces significantly due to low spiking activity (=0.0092). Figure 16B shows the power distribution of the synaptic cores in the training mode. It shows the much higher power consumption compared to the inference mode, mainly because of the complex weight update module (Kim et al., 2020).

The central STDP controller consists of 32 × 128 SRAM, the control logic, and the *V*_mem_ Comparator. The read energy is 1.14 pJ, and the write energy is 3.09 pJ. The bus-width is 32 bits. It also operates at 1 GHz by following the Synapse Core's clock frequency. Overall, the central STDP controller has a much smaller area (0.025 *mm*^2^) and power (0.141 mW during inference and 0.154 mW during learning).

### 4.6. Comparison With Prior Works

Table 3 shows the comparison of MONETAwith a set of recent SNN accelerators (Buhler et al., 2017; Chen et al., 2019; Park et al., 2019; Chuang et al., 2020). Note that all designs use different SNN architectures for evaluation, and most of the prior designs considered MNIST as the dataset while our work is evaluated on MNIST CIFAR-10 and CIFAR-100. Our design supports STDP learning (fully for the homogeneous network and partially for the hybrid network) and inference.

**Table 3:** Comparison with other works.

**Reference**	**This work**				**DAC‘20**	**ISSCC‘19**	**JSSC‘19**	**VLSI‘17**

	**Homogeneous**		**Hybrid**					
	**Inference**	**Learning**	**Inference**	**Learning**				
Technology (nm)	65				90	65	10	40
Algorithm	ConvSNN				ConvSNN	SNN	SNN	SNN
On-chip Training	Yes				No	Yes	Yes	Yes
Voltage (V)	1.0				1.0	0.8	0.9	0.9
Frequency (MHz)	1,000				100	20	506	250
Synapse Bits	8				1	7	7	4
Area (mm^2^)	11.155		22.23		2.07	10.08	1.72	1.31
TOPS/mm^2^	0.207	0.197	0.207	0.197	0.312	0.008	0.015	0.227
Power (mW)	123.28	303.52	246.56	423.83	45.71	23.6	208.3	87
TOPS/W	18.69	7.25	18.69	10.41	14.1	3.42	0.12	3.43

Our throughput is higher than prior works mainly due to highly parallel in-memory computation, as well as higher frequency (1 GHz) of operation. The PIM architecture eliminates the arithmetic computation units used in prior designs leading to a much higher operating speed at similar voltage. Thanks to the PIM architecture, MONETAshows higher compute density (TOPS/mm^2^) compared to the prior works using similar bit-precision. We observe similar area efficiency compared to 4-bit precision-based SNN in 40 nm CMOS, even though our design is realized in 65 nm CMOS. However, compared to the binary SNN design we observe 33% lower area efficiency (note, the binary SNN was implemented in 90 nm CMOS). Further, we observe a higher power efficiency compared to other designs during inference and learning. This is mainly because the PIM-based operation naturally translates the sparsity in neuron firing to power reduction as discussed before.

## 5. Conclusion

This article presents a PIM-based hybrid ConvSNN acceleration platform with an on-chip STDP based weight update. We present an optimized data flow for sequential processing of input feature maps to reduce off-chip data movement while ensuring learning accuracy of the STDP process. The algorithmic simulations show comparable accuracy for MNIST and CIFAR-10 dataset to a pure software implementation. We also show the hybrid architecture and the opportunity of the supervised-unsupervised flexible weight architecture with on-line learning. The power and throughput analysis using 65 nm CMOS physical design show high throughput and energy efficiency. The programming model and compiler infrastructure necessary to map an arbitrary ConvSNN in MONETAis important future work.

## Data Availability Statement

Publicly available datasets were analyzed in this study. This data can be found at: MNIST: http://yann.lecun.com/exdb/mnist/; https://www.cs.toronto.edu/~kriz/cifar.html.

## Author Contributions

DK developed the main concepts and algorithm, generated the RTL design and layout, and performed all hardware analysis. BC and XS developed the algorithm for a hybrid network and performed the software simulation for accuracy analysis. All authors assisted in developing the concept and writing this article. All authors contributed to the article and approved the submitted version.

## Funding

This research was supported by the DARPA ERI 3DSoC Program under Award HR001118C0096.

## Author Disclaimer

The views and conclusions included in this article are those of the authors and should not be interpreted as representing the official policies, either expressed or implied, of DARPA or the U.S. Government.

## Conflict of Interest

The authors declare that the research was conducted in the absence of any commercial or financial relationships that could be construed as a potential conflict of interest.

## Publisher's Note

All claims expressed in this article are solely those of the authors and do not necessarily represent those of their affiliated organizations, or those of the publisher, the editors and the reviewers. Any product that may be evaluated in this article, or claim that may be made by its manufacturer, is not guaranteed or endorsed by the publisher.

## References

[B1] Akopyan F. Sawada J. Cassidy A. Alvarez-Icaza R. Arthur J. Merolla P.. (2015). Truenorth: Design and tool flow of a 65 mw 1 million neuron programmable neurosynaptic chip. IEEE Trans. Comput.-Aided Des. Integr. Circ. Syst. 34, 1537–1557. 10.1109/TCAD.2015.2474396

[B2] Bi G.-Q. Poo M.-M. (1998). Synaptic modifications in cultured hippocampal neurons: dependence on spike timing, synaptic strength, and postsynaptic cell type. J. Neurosci. 18, 10464–10472. 9852584 10.1523/JNEUROSCI.18-24-10464.1998 PMC6793365

[B3] Buhler F. N. Brown P. Li J. Chen T. Zhang Z. Flynn M. P. (2017). “A 3.43tops/w 48.9pj/pixel 50.1nj/classification 512 analog neuron sparse coding neural network with on-chip learning and classification in 40nm cmos,” in 2017 Symposium on VLSI Circuits (Kyoto), C30–C31.

[B4] Cao Y. Chen Y. Khosla D. (2015). Spiking deep convolutional neural networks for energy-efficient object recognition. Int. J. Comput. Vis. 113, 54–66. 10.1007/s11263-014-0788-3

[B5] Chakraborty B. She X. Mukhopadhyay S. (2021). A fully spiking hybrid neural network for energy-efficient object detection. IEEE Trans. Image Process. 30, 9014–9029. 10.1109/TIP.2021.3122092 34705647

[B6] Chen G. K. Kumar R. Sumbul H. E. Knag P. C. Krishnamurthy R. K. (2019). A 4096-neuron 1m-synapse 3.8-pj/sop spiking neural network with on-chip stdp learning and sparse weights in 10-nm finfet cmos. IEEE J. Solid-State Circ. 54, 992–1002. 10.1109/JSSC.2018.2884901

[B7] Chen Y. Krishna T. Emer J. Sze V. (2017). Eyeriss: an energy-efficient reconfigurable accelerator for deep convolutional neural networks. IEEE J. Solid-State Circ. 52, 127–138. 10.1109/JSSC.2016.2616357

[B8] Chi P. Li S. Xu C. Zhang T. Zhao J. Liu Y.. (2016). “Prime: a novel processing-in-memory architecture for neural network computation in reram-based main memory,” in Proceedings of the 43rd International Symposium on Computer Architecture ISCA '16 (Seoul: IEEE Press), 27–39.

[B9] Chuang P. Y. Tan P.-Y. Wu C.-W. Lu J.-M. (2020). “A 90nm 103.14 tops/w binary-weight spiking neural network cmos asic for real-time object classification,” in 2020 57th ACM/IEEE Design Automation Conference (DAC) (San Francisco, CA), 1–6.

[B10] Davies M. Srinivasa N. Lin T.-H. Chinya G. Cao Y. Choday S. H.. (2018). Loihi: a neuromorphic manycore processor with on-chip learning. IEEE Micro 38, 82–99. 10.1109/MM.2018.112130359

[B11] Deng J. Dong W. Socher R. Li L.-J. Li K. Fei-Fei L. (2009). “Imagenet: a large-scale hierarchical image database,” in 2009 IEEE Conference on Computer Vision and Pattern Recognition (Miami, FL: IEEE), 248–255.

[B12] Deng L. Wang G. Li G. Li S. Liang L. Zhu M.. (2020). Tianjic: aunified and scalable chip bridging spike-based and continuous neural computation. IEEE J. Solid-State Circ. 55, 2228–2246. 10.1109/JSSC.2020.2970709

[B13] Diehl P. U. Cook M. (2015). Unsupervised learning of digit recognition using spike-timing-dependent plasticity. Front. Comput. Neurosci. 9, 99. 10.3389/fncom.2015.00099 26941637 PMC4522567

[B14] Diehl P. U. Neil D. Binas J. Cook M. Liu S.-C. Pfeiffer M. (2015). “Fast-classifying, high-accuracy spiking deep networks through weight and threshold balancing,” in 2015 International Joint Conference on Neural Networks (IJCNN) (Killarney: IEEE), 1–8.

[B15] Gerstner W. Kistler W. (2002). Spiking Neuron Models: Single Neurons, Populations, Plasticity. Camebridge: Cambridge University Press.

[B16] He K. Zhang X. Ren S. Sun J. (2016). “Deep residual learning for image recognition,” in Proceedings of the IEEE Conference on Computer Vision and Pattern Recognition (Las Vegas, NV), 770–778.

[B17] Imani M. Gupta S. Kim Y. Rosing T. (2019). “Floatpim: in-memory acceleration of deep neural network training with high precision,” in 2019 ACM/IEEE 46th Annual International Symposium on Computer Architecture (ISCA) (Phoenix, AZ), 802–815.

[B18] Kheradpisheh S. R. Ganjtabesh M. Thorpe S. J. Masquelier T. (2018). STDP-based spiking deep convolutional neural networks for object recognition. Neural Netw. 99, 56–67. 10.1016/j.neunet.2017.12.005 29328958

[B19] Kim D. She X. Rahman N. M. Chekuri V. C. K. Mukhopadhyay S. (2020). Processing-in-memory-based on-chip learning with spike-time-dependent plasticity in 65-nm cmos. IEEE Solid-State Circ. Lett. 3, 278–281. 10.1109/LSSC.2020.3013448

[B20] Kim S. Park S. Na B. Yoon S. (2020). “Spiking-yolo: spiking neural network for energy-efficient object detection,” in Proceedings of the AAAI Conference on Artificial Intelligence (New York, NY), vol. 34, 11270–11277.

[B21] Ledinauskas E. Ruseckas J. Juršėnas A. Buračas G. (2020). Training deep spiking neural networks. arXiv preprint arXiv:2006.04436. 10.48550/ARXIV.2006.04436

[B22] Lee C. Panda P. Srinivasan G. Roy K. (2018). Training deep spiking convolutional neural networks with stdp-based unsupervised pre-training followed by supervised fine-tuning. Front. Neurosci. 12, 435. 10.3389/fnins.2018.00435 30123103 PMC6085488

[B23] Lee C. Sarwar S. S. Panda P. Srinivasan G. Roy K. (2020). Enabling spike-based backpropagation for training deep neural network architectures. Front Neurosci. 14, 119. 10.3389/fnins.2020.00119 32180697 PMC7059737

[B24] Lee C. Srinivasan G. Panda P. Roy K. (2019). Deep spiking convolutional neural network trained with unsupervised spike-timing-dependent plasticity. IEEE Trans. Cogn. Develop. Syst. 11, 384–394. 10.1109/TCDS.2018.2833071

[B25] Long Y. (2019). A ferroelectric fet-based processing-in-memory architecture for dnn acceleration. IEEE J. Exp. Solid-State Comput. Dev. Circ. 5, 113–122. 10.1109/JXCDC.2019.2923745

[B26] Long Y. Lee E. Kim D. Mukhopadhyay S. (2020). “Q-pim: a genetic algorithm based flexible dnn quantization method and application to processing-in-memory platform,” in 2020 57th ACM/IEEE Design Automation Conference (DAC) (San Francisco, CA), 1–6.

[B27] Maass W. (1997). Networks of spiking neurons: the third generation of neural network models. Neural Netw. 10, 1659–1671.

[B28] Miquel J. R. Tolu S. Schöller F. E. Galeazzi R. (2021). Retinanet object detector based on analog-to-spiking neural network conversion. arXiv preprint arXiv:2106.05624. 10.48550/ARXIV.2106.05624

[B29] Narayanan S. Taht K. Balasubramonian R. Giacomin E. Gaillardon PE. (2020). “Spinalflow: an architecture and dataflow tailored for spiking neural networks,” in 2020 ACM/IEEE 47th Annual International Symposium on Computer Architecture (ISCA) (Valencia), 349–362.

[B30] Neftci E. O. Mostafa H. Zenke F. (2019). Surrogate gradient learning in spiking neural networks: bringing the power of gradient-based optimization to spiking neural networks. IEEE Signal Process. Mag. 36, 51–63. 10.1109/MSP.2019.2931595

[B31] Panda P. Aketi S. A. Roy K. (2020). Toward scalable, efficient, and accurate deep spiking neural networks with backward residual connections, stochastic softmax, and hybridization. Front. Neurosci. 14, 653. 10.3389/fnins.2020.00653 32694977 PMC7339963

[B32] Park J. Lee J. Jeon D. (2019). “7.6 a 65nm 236.5nj/classification neuromorphic processor with 7.5energy overhead on-chip learning using direct spike-only feedback,” in 2019 IEEE International Solid- State Circuits Conference - (ISSCC) (San Francisco, CA), 140–142.

[B33] Peng X. Huang S. Jiang H. Lu A. Yu S. (2021). DNN+NeuroSim V2.0: An end-to-end benchmarking framework for compute-in-memory accelerators for on-chip training. IEEE Trans. Comput. Aid. D. Integ. Circui.t Syst. 40, 2306–2319. 10.1109/TCAD.2020.3043731

[B34] Pfeiffer M. Pfeil T. (2018). Deep learning with spiking neurons: opportunities and challenges. Front. Neurosci. 12, 774. 10.3389/fnins.2018.00774 30410432 PMC6209684

[B35] Sengupta A. Ye Y. Wang R. Liu C. Roy K. (2019). Going deeper in spiking neural networks: vgg and residual architectures. Front. Neurosci. 13, 95. 10.3389/fnins.2019.00095 30899212 PMC6416793

[B36] Seo J. Brezzo B. Liu Y. Parker B. D. Esser S. K. Montoye R. K.. (2011). “A 45nm cmos neuromorphic chip with a scalable architecture for learning in networks of spiking neurons,” in 2011 IEEE Custom Integrated Circuits Conference (CICC) (San Jose, CA), 1–4.

[B37] Shafiee A. Nag A. Muralimanohar N. Balasubramonian R. Paul Strachan J. Hu M.. (2016). “Isaac: a convolutional neural network accelerator with in-situ analog arithmetic in crossbars,” in Proceedings of the 43rd International Symposium on Computer Architecture ISCA '16 (Seoul: IEEE Press), 14–26.

[B38] She X. Long Y. Kim D. Mukhopadhyay S. (2021). Scienet: deep learning with spike-assisted contextual information extraction. Pattern Recogn. 118, 108002. 10.1016/j.patcog.2021.108002

[B39] She X. Long Y. Mukhopadhyay S. (2019). “Fast and low-precision learning in gpu-accelerated spiking neural network,” in 2019 Design, Automation & Test in Europe Conference & Exhibition (DATE) (Florence: IEEE), 450–455.

[B40] She X. Saha P. Kim D. Long Y. Mukhopadhyay S. (2020). “Safe-dnn: a deep neural network with spike assisted feature extraction for noise robust inference,” in 2020 International Joint Conference on Neural Networks (IJCNN) (Glasgow: IEEE), 1–8.

[B41] Simonyan K. Zisserman A. (2014). Very deep convolutional networks for large-scale image recognition. arXiv preprint arXiv:1409.1556. 10.48550/ARXIV.1409.1556

[B42] Singh S. Sarma A. Jao N. Pattnaik A. Lu S. Yang K.. (2020). “Nebula: a neuromorphic spin-based ultra-low power architecture for snns and anns,” in 2020 ACM/IEEE 47th Annual International Symposium on Computer Architecture (ISCA) (Valencia), 363–376.

[B43] Srinivasan G. Panda P. Roy K. (2018). Stdp-based unsupervised feature learning using convolution-over-time in spiking neural networks for energy-efficient neuromorphic computing. ACM J. Emerg. Technol. Comput. Syst. (JETC) 14, 1–12. 10.1145/3266229

[B44] Sze V. Chen Y.-H. Yang T.-J. Emer J. S. (2020). “Efficient processing of deep neural networks,” in Synthesis Lectures on Computer Architecture San Rafael, CA: Morgan and Claypool, vol. 15. 1–341.

[B45] Tavanaei A. Maida A. S. (2016). Bio-inspired spiking convolutional neural network using layer-wise sparse coding and stdp learning. arXiv [Preprint]. arXiv: 1611.03000. Available online at: https://arxiv.org/pdf/1611.03000.pdf

[B46] Wang G. Ma S. Wu Y. Pei J. Zhao R. Shi L. (2021). End-to-end implementation of various hybrid neural networks on a cross-paradigm neuromorphic chip. Front. Neurosci. 15, 45. 10.3389/fnins.2021.615279 33603643 PMC7884322

[B47] Wu Y. Deng L. Li G. Zhu J. Shi L. (2018). Spatio-temporal backpropagation for training high-performance spiking neural networks. Front. Neurosci. 12, 331. 10.3389/fnins.2018.00331 29875621 PMC5974215

